# Supplementary material for: Pathogenic variants reveal candidate genes for prostate cancer germline testing for men of African ancestry
Source: Nat Commun. 2025 Oct 2;16:8799. doi: 10.1038/s41467-025-63865-6 (PMC12491615; doi:10.1038/s41467-025-63865-6)
Supplement: Supplementary file 1 — Supplementary information [file 41467_2025_63865_MOESM1_ESM.pdf]

Pathogenic variants reveal candidate genes for prostate cancer germline testing for men of African ancestry

Gheybi K, et al.

DATA SUPPLEMENT

Supplementary Tables

Table S1. Whole germline sequenced prostate cancer patient resource data available to this study and defined by patient ancestry.

Patient source and country	African (n = 217)		European (n = 959)	
	**EGA Data source**	**Number**	**EGA Data Source**	**Number**
**SAPCS (n = 186)**				
South Africa (Jaratlerdsiri *et al.*, 2022)	EGAD00001009067	116	NA	0
South Africa (This Study)	EGAD50000001626	70	NA	0
	**Total SAPCS**	**186**	**NA**	**NA**
**PPCG (n = 1,090)**				
Canadian PCa Genome Network	EGAD00001004170	11	EGAD00001004170	292
CRUK-ICGC Prostate Group UK	EGAC00001000852	10	EGAC00001000852	217
French/Caribbean ICGC PCa Group	EGAD00001003835	10	EGAD00001003835	15
Germany ICGC PCa Group	NA	0	EGAD00001005997	238
Melbourne Research Group Australia	NA	0	EGAD00001004182	144
Sydney St Vincents/Garvan Australia	NA	0	EGAD00001009066	53
	**Total PPCG**	**31**	**Total PPCG**	**959**

Abbreviations: CRUK, Cancer Research United Kingdom; EGA, European Genome-Phenome Achieve; ICGC, International Cancer Genome Consortium; PCa, Prostate Cancer; NA, Not Applicable; PPCG, Pan Prostate Cancer Group; SAPCS, Southern African Prostate Cancer Study

Table S2. African ancestral patient characteristics (n = 217) at diagnosis (SAPCS) or surgery (PPCG).

		**SAPCS (Southern African)**	**PPCG (Global African)**	**Overall**
Patients, N		186	31	217
Mean age (SD)		66.7 (8.5) [range 43-99]	64.8 (7.3) [range 45-77]	66.4 (8.3)
Mean PSA (SD)		233.6 (637.6) [range 1-4841]	60.8 (212.3) [range 5-1150]	206.2 (595.3)
ISUP GG[a]	1	33 (17.7%)	1 (3.2%)	34 (15.3%)
	2	31 (16.7%)	16 (51.6%)	47 (21.2%)
	3	20 (10.7%)	8 (25.8%)	28 (13.1%)
	4/5	99 (53.2%)	6 (19.4%)	106 (48.7%)

[a]Three of the South African patients had missing information on ISUP grade.

Abbreviations: ISUP GG, International Society of Urological Pathology Grade Group; PPCG, Pan Prostate Cancer Group; PSA, Prostate Specific Antigen; SAPCS, Southern African Prostate Cancer Study; SD, Standard Deviation.

Table S3. Rare potentially pathogenic variants (PPVs) shared between the 959 PPCG White and the 217 Black prostate cancer (PCa) patients from this study.

Gene	Chr: position; allele change	rsID	AA change	White PPCG (n=959)	Black (this study, n=217)
ABCA4	chr1:94098928:G/A	rs61750200	R212C	1	1
ETFDH	chr4:158708445:CAT/C	rs767795266	C592X	1	1
RAD50	chr5:132595759T/TA[a]	rs397507178	E723fs	1	1
DAAM2	chr6:39879377:C/A	rs150676991	P582H	2	1
ACHE	chr7:100892545:G/A	rs1399936353	R448X	1	1
LRSAM1	chr9:127496079:CA/A	rs747130246	D607fs	1	1
DHTKD1	chr10:12097688:C/T	rs201369986	R455X	1	1
AAAS	chr12:53321423:G/T	rs121918549	Q15K	1	1
SGSH	chr17:80217061:G/A	rs104894636	R74C	1	1
U2AF1	chr21:43094667:T/G	rs371246226	Q157P	1	1
AIRE	chr21:44289773:C/T	rs121434254	R257X	1	1
CHEK2	chr22:28734439:G/A[a]	rs587781269	R283X	1	1
NCF4	chr22:36875833:C/T	rs201021542	R270X	1	1

[a] Known to PCa

Abbreviations: AA, amino acid; PPCG, Pan Prostate Cancer Group

Table S4. Potentially pathogenic variants (PPVs) shared between the 3,209 MGRB white healthy individuals and the 217 Black prostate cancer patients in this study.

Gene	Chr position; allele change	Rs ID	White MGRB Allele count (n = 3,209)	White PPCG Allele count (n = 959)	Black PCa Allele count (n = 217)
ALG6	chr1:63402348:G/A	rs199682486	11	0	1
ABCA4	chr1:94098928:G/A	rs61750200	1	1	1
ADCY10	chr1:167824528:G/A	rs146725782	2	0	1
PRSS56	chr2:232523817:A/AC	rs730882064	3	0	1
DAAM2	chr6:39879377:C/A	rs150676991	4	2	1
PKHD1	chr6:52048558:G/A	rs398124478	1	0	1
STAG3	chr7:100204096:C/T	rs764841861	1	0	1
GDF6	chr8:96160524:C/G	rs397514725	1	0	1
LRSAM1	chr9:127496079:CA/C	rs747130246	1	1	1
RET	chr10:43119548:G/A	rs79658334	1	0	1
NANOS1	chr10:119030300:CCCG CCGCCG/CCCGCCG	rs538539239	8	0	1
DHTKD1	chr10:12097688:C/T	rs201369986	2	0	1
AAAS	chr12:53321423:G/T	rs121918549	1	1	1
TGM1	chr14:24259744:C/T	rs143473912	2	0	2
AGRP	chr16:67483042:C/T	rs5030980	259	7	11
FKBP10	chr17:41819306:T/TC	rs137853883	2	0	3
SGSH	chr17:80217061:G/A	rs104894636	1	1	1
AIRE	chr21:44289773:C/T	rs121434254	2	1	1
STAG2	chrX:124066174:G/T	rs1603095192	1	0	1

Abbreviations: chr, chromosome; MGRB, Medical Genome Reference Biobank; PCa, prostate cancer; PPCG, Pan Prostate Cancer Group

Supplementary Figure

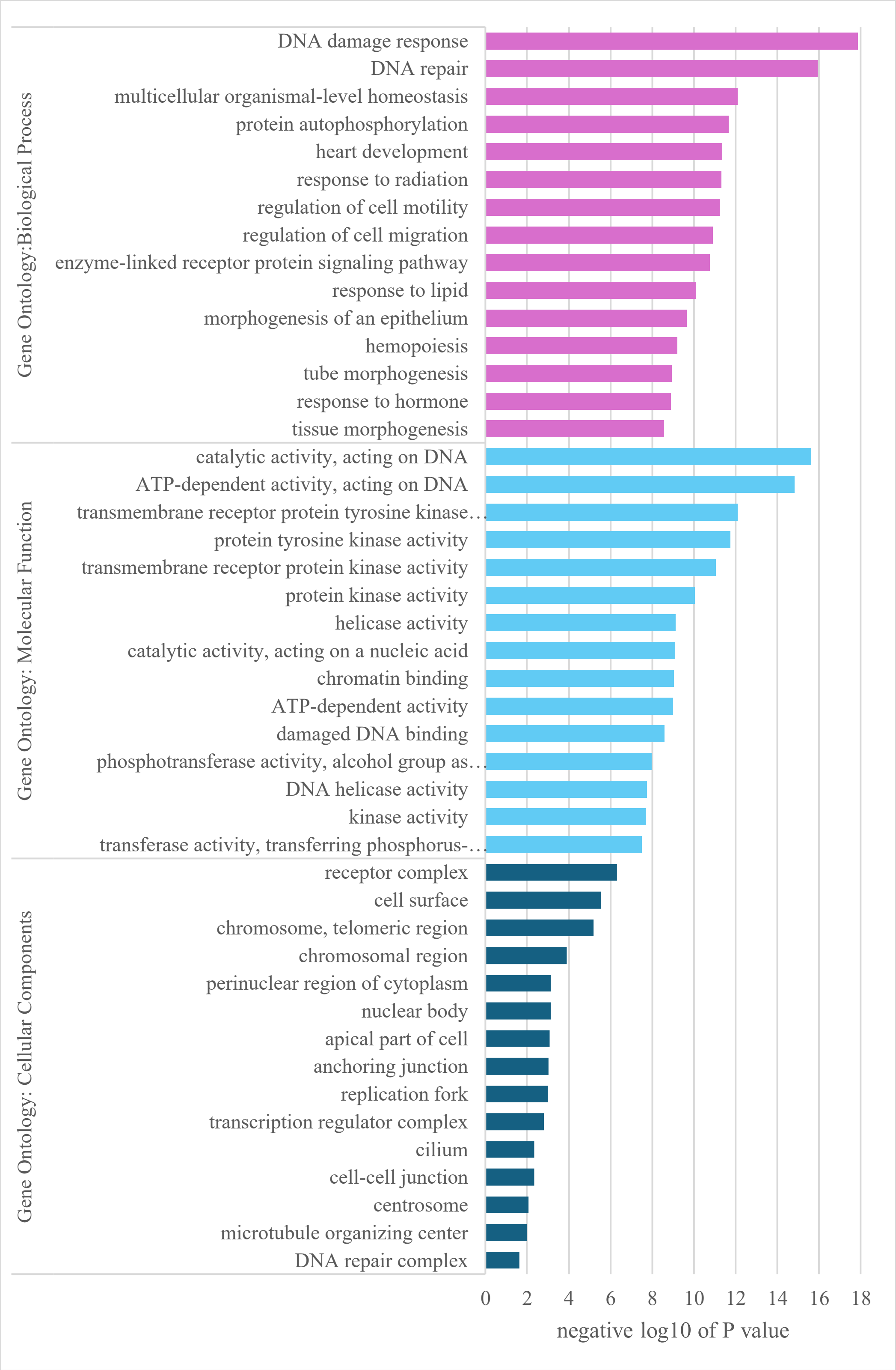

Figure S1. Gene ontology: most significantly enriched pathways in genes harbouring PPV/POV among 217 African prostate cancer patients
