# Supplementary material for: Pathogenic variants reveal candidate genes for prostate cancer germline testing for men of African ancestry
Source: Nat Commun. 2025 Oct 2;16:8799. doi: 10.1038/s41467-025-63865-6 (PMC12491615; doi:10.1038/s41467-025-63865-6)
Supplement: Supplementary file 3 — Reporting Summary [file 41467_2025_63865_MOESM3_ESM.pdf]

nature portfolio

Corresponding author(s): Prof Vanessa M. Hayes

Last updated by author(s): 16 July 2025

# Reporting Summary

Nature Portfolio wishes to improve the reproducibility of the work that we publish. This form provides structure for consistency and transparency in reporting. For further information on Nature Portfolio policies, see our Editorial Policies and the Editorial Policy Checklist.

## Statistics

For all statistical analyses, confirm that the following items are present in the figure legend, table legend, main text, or Methods section.

| n/a | Confirmed | |
|---|---|---|
| ☐ | ☒ | The exact sample size (*n*) for each experimental group/condition, given as a discrete number and unit of measurement |
| ☒ | ☐ | A statement on whether measurements were taken from distinct samples or whether the same sample was measured repeatedly |
| ☒ | ☐ | The statistical test(s) used AND whether they are one- or two-sided<br>*Only common tests should be described solely by name; describe more complex techniques in the Methods section.* |
| ☒ | ☐ | A description of all covariates tested |
| ☒ | ☐ | A description of any assumptions or corrections, such as tests of normality and adjustment for multiple comparisons |
| ☒ | ☐ | A full description of the statistical parameters including central tendency (e.g. means) or other basic estimates (e.g. regression coefficient) AND variation (e.g. standard deviation) or associated estimates of uncertainty (e.g. confidence intervals) |
| ☒ | ☐ | For null hypothesis testing, the test statistic (e.g. *F*, *t*, *r*) with confidence intervals, effect sizes, degrees of freedom and *P* value noted<br>*Give P values as exact values whenever suitable.* |
| ☒ | ☐ | For Bayesian analysis, information on the choice of priors and Markov chain Monte Carlo settings |
| ☒ | ☐ | For hierarchical and complex designs, identification of the appropriate level for tests and full reporting of outcomes |
| ☒ | ☐ | Estimates of effect sizes (e.g. Cohen's *d*, Pearson's *r*), indicating how they were calculated |

*Our web collection on statistics for biologists contains articles on many of the points above.*

## Software and code

Policy information about availability of computer code

| | |
|---|---|
| Data collection | Software and scripts for DNA sequence read data collection, and the scripts for sequence read alignment and quality control are available on GitHub (https://github.com/Sydney-Informatics-Hub/Bioinformatics). |
| Data analysis | As above |

For manuscripts utilizing custom algorithms or software that are central to the research but not yet described in published literature, software must be made available to editors and reviewers. We strongly encourage code deposition in a community repository (e.g. GitHub). See the Nature Portfolio guidelines for submitting code & software for further information.

## Data

Policy information about availability of data

All manuscripts must include a data availability statement. This statement should provide the following information, where applicable:

- Accession codes, unique identifiers, or web links for publicly available datasets
- A description of any restrictions on data availability
- For clinical datasets or third party data, please ensure that the statement adheres to our policy

Data availability
Access to published whole genome sequence data published in Jaratlerdsiri et al was made available via Data Access Committee (DAC) approval as outlined under the European Genome-Phenome Archive (EGA) [https://ega-archive.org] project-specific access policies under overarching study EGAS00001006425 [https://www.ega-archive.org/studies/EGAS00001006425], which includes the Southern African Prostate Cancer Study (SAPCS) Dataset at EGAD00001009067 [https://

www.ega-archive.org/datasets/EGAD00001009067] and as part of the PPCG cohort the Garvan/St Vincent's Prostate Cancer or Sydney Database at EGAD00001009066 [https://www.ega-archive.org/datasets/EGAD00001009066], while additional PPCG Datasets are summarised in Table S1 and include Canadian PCa Genome Network [https://ega-archive.org/datasets/EGAD00001004170], CRUK-ICGC Prostate Group UK [https://ega-archive.org/datasets/EGAC00001000852], French/Caribbean ICGC PCa Group [https://ega-archive.org/datasets/EGAD00001003835], Germany ICGC PCa Group [https://ega-archive.org/datasets/EGAD00001005997], and Melbourne Research Group Australia [https://ega-archive.org/datasets/EGAD00001004182]. MGRB data is available as defined by study EGAS00001003511 [https://www.ega-archive.org/studies/EGAS00001003511] and dataset EGAD00001005228 [https://ega-archive.org/datasets/EGAD00001005228]. The additional 70 SAPCS germline whole genome data has been deposited under the overarching study EGAS50000001132 [https://submission.ega-archive.org/submissions/EGA50000001053] and dataset EGAD50000001626 [https://submission.ega-archive.org/submissions/EGA50000001053/datasets]. Additional variant and annotation data for the Black PCa patient, European PPCG patient, African and healthy control populations study are available within the main text and supplementary information.

Data availability statement. Access to the SAPCS sequencing data may be requested via the SAPCS DAC and will be made available to researchers with appropriate feasibility and corresponding ethics approvals to ensure the safeguarding of patient genomic information (contact V.M.H. or M.S.R.B.). Restrictions include (i) No transfer to third parties allowed, (ii) acknowledgment of the SAPCS in publications/presentations, (iii) a report of the results of the research to be provided to DAC after completion (or when requested), (iv) researchers cannot utilize the data for commercial purposes or any other purposes not approved by the DAC, and (v) approval will not be given that excludes other researchers from accessing data. Data currently being used for capacity building in under-resourced studies across Sub-Saharan Africa will be given priority and at times may be granted time-limited exclusive rights for no more than a two-year period.

Supporting data. SNVs and indels data supporting the findings of this study are available within the main text, Supplementary information and source data. Previously published SNV and indel sites and their minor allele frequencies are available in the dbSNP [https://www.ncbi.nlm.nih.gov/snp/], and gnomAD databases [https://gnomad.broadinstitute.org/]. Gene regions are available in the ENSEMBL database [https://www.ensembl.org], and DDR gene list is available at GSEA [https://www.gsea-msigdb.org/gsea/msigdb/human/geneset/HALLMARK_DNA_REPAIR.html].

# Research involving human participants, their data, or biological material

Policy information about studies with human participants or human data. See also policy information about sex, gender (identity/presentation), and sexual orientation and race, ethnicity and racism.

Reporting on sex and gender

Prostate cancer is a male only condition. The term male or men (referrring to biological gender due to a presence of a prostate gland) is used through out. Self-identified sex and gender was not recorded or reported.

Reporting on race, ethnicity, or other socially relevant groupings

Race/ethnicity, defined in our study by ethno-linguistic identifiers was self-reported and for the SAPCS participants were grouped as Black South African and EAPCS control participants as Kenyans. Additionally, patient ancestry was determined using genetics distinguishing African from non-African or European ancestral genetic fractions.

Population characteristics

PCa patients. The 217 African ancestral participants were recruited either at PCa diagnosis from a participating SAPCS urology clinic in South Africa or at radical prostatectomy from a participating PPCG member site. Study inclusion was based on a histopathological confirmation of PCa defined as a Gleason score or an International Society of Urological Pathology Grade Group (ISUP) and a self-reported and/or genetically predicted African ancestry. For the SAPCS, 186 men self-identifying as Black South African were selected for whole genome interrogation, including both published (n=115)18 and unpublished data (n=71). The additional PCa patients represented self-identified Black South Africans recruited at research hubs for the TARGET Africa and/or HEROIC PCaPH Africa1K US-DoD-funded projects, which included Dr George Mukhari Academic Hospital of the Sefako Makgatho Health Sciences University, an urban hub in the province of Gauteng, or at Tshilidzini Hospital, an approved University of Pretoria research hub, within the rural province of Limpopo. Conversely, the PPCG includes whole genome data for 959 PCa cases sourced from Canada (n=303), Germany (n=238), United Kingdom (n=226) 64,65, Australia (n=143 Melbourne, 53 Sydney)18, and France (n=25), of which 31 (3.1%), including 11 Canadians, 10 British and 10 French Caribbeans, reported African ancestry.

African controls. The HEROIC PCaPH Africa1K has access to 49 southern Africans self-identified from one or more southern Bantu ethno-linguistic group and recruited as part of the SAPCS, and 40 east Africans self-identified from either an eastern Bantu or Nilotic ethno-linguistic group via the EAPCS. Participation as a population-matched study control included two-generational African ethno-linguistic identity, being less than 50 years of age, no PCa or any cancer diagnosis, and unlike our case cohort, representing any self-reported gender. Having undergone deep whole genome sequencing (unpublished), provided the background for targeted candidate gene interrogation for population relevant MAFs.

Healthy controls. The MGRB samples were gathered from 3,209 white Australian individuals aged 75 years or older with no known metabolic illnesses including hypertension, cancer, or dementia. WGS of the samples was performed on Illumina HiSeq X sequencers generating a median coverage of 37.31X (range 21.95 to 44.12X). Mapping was built on GRCh37 and variant calling was performed following GATK best practices as previously described.

Recruitment

See above. Recruitment for patients was limited to participating urology clinics. African control recruitment was biased to female participants, men without prostate cancer and younger aged participants. PPCG recruitment was as per original study, as with the healthy controls.

Ethics oversight

Patients and population-representative controls provided informed consent to participate in the study and were recruited as part of the SAPCS (patients and controls) or East African Prostate Cancer Study (EAPCS, controls only). For the SAPCS, study approval was granted by the University of Pretoria Faculty of Human Research Ethics Committee (HREC #43/2010, including US Federal-wide Assurance FWA00002567 and IRB00002235 IORG0001762) in South Africa, with additional Institutional Review Board (IRB) approval granted by the Human Research Protection Office (HRPO) of the US Army Medical Research and Development Command (E02371.2a TARGET Africa; E03333.1a and E05986.1a HEROIC PCaPH Africa1K). For the EAPCS, study approval was granted by the Kenyatta National Hospital (KNH) and University of Nairobi (UON) Ethics Research Committee (ERC) in Kenya (KNH/UON-ERC P637/07/2019), with additional IRB approval granted by the US Army Medical Research and

Development Command HRPO (E03347.1b and E05987.1a HEROIC PCaPH Africa1K). Samples (whole blood) were shipped to the University of Sydney in accordance with institutional Material Transfer Agreements (MTAs) and including for the SAPCS under a Republic of South Africa Department of Health Export Permit (National Health Act 2003; J1/2/4/2), while data sharing includes is made possible by a full-executed inter-institutional Collaborative Research Agreement (CRA) between the HEROIC PCaPH Africa1K study leads including the University of Sydney (Australia), University of Pretoria (South Africa), University of Nairobi (Kenya) and University of Chicago (U.S.A.). Molecular genetic research for patients from the SAPCS bioresource was approved by the St. Vincent's Hospital Human Research Ethics Committee in Sydney in Australia (#SVH15/227), with additional IRB approval granted by the US Army Medical Research and Development Command HRPO (E02371 TARGET Africa; E03280.1a and E05984.1a HEROIC PCaPH Africa1K). As an International Cancer Genome Consortium (ICGC) member, the PPCG collection is subject to the standards of ethical consent. Country specific IRB approvals, which included for Australian samples from Melbourne (Epworth Health 34506; Melbourne Health 2019.058) and Sydney (St Vincent's HREC #SVH/12/231).

Note that full information on the approval of the study protocol must also be provided in the manuscript.

# Field-specific reporting

Please select the one below that is the best fit for your research. If you are not sure, read the appropriate sections before making your selection.

☒ Life sciences ☐ Behavioural & social sciences ☐ Ecological, evolutionary & environmental sciences

For a reference copy of the document with all sections, see nature.com/documents/nr-reporting-summary-flat.pdf

# Life sciences study design

All studies must disclose on these points even when the disclosure is negative.

| | |
|---|---|
| Sample size | The 217 African ancestral participants were recruited either at PCa diagnosis from a participating SAPCS urology clinic in South Africa or at radical prostatectomy from a participating PPCG member site. Sample size was solely dependent on maximising the number of African ancestral patients to achieve the largest African-specific study of whole germline pathogenic variant interrogation. |
| Data exclusions | No data was excluded, however, inclusion criteria were based on: (i) African ancestry (ii) clinicopathologically confirmed prostate cancer and (iii) patient consent for whole genome interrogation. |
| Replication | As the largest resource for the region, patient matched replication data is not available. |
| Randomization | NA |
| Blinding | While data generation (sequencing) is blinded to patient source or ancestry, downstream analyses was subject to pateint having sufficient African ancestry as per inclusion criteria stated above. |

# Reporting for specific materials, systems and methods

We require information from authors about some types of materials, experimental systems and methods used in many studies. Here, indicate whether each material, system or method listed is relevant to your study. If you are not sure if a list item applies to your research, read the appropriate section before selecting a response.

## Materials & experimental systems

| n/a | Involved in the study | |
|---|---|---|
| ☒ | ☐ | Antibodies |
| ☒ | ☐ | Eukaryotic cell lines |
| ☒ | ☐ | Palaeontology and archaeology |
| ☒ | ☐ | Animals and other organisms |
| ☒ | ☐ | Clinical data |
| ☒ | ☐ | Dual use research of concern |
| ☒ | ☐ | Plants |

## Methods

| n/a | Involved in the study | |
|---|---|---|
| ☒ | ☐ | ChIP-seq |
| ☒ | ☐ | Flow cytometry |
| ☒ | ☐ | MRI-based neuroimaging |

## Plants

Seed stocks

*Report on the source of all seed stocks or other plant material used. If applicable, state the seed stock centre and catalogue number. If plant specimens were collected from the field, describe the collection location, date and sampling procedures.*

Novel plant genotypes

*Describe the methods by which all novel plant genotypes were produced. This includes those generated by transgenic approaches, gene editing, chemical/radiation-based mutagenesis and hybridization. For transgenic lines, describe the transformation method, the number of independent lines analyzed and the generation upon which experiments were performed. For gene-edited lines, describe the editor used, the endogenous sequence targeted for editing, the targeting guide RNA sequence (if applicable) and how the editor was applied.*

Authentication

*Describe any authentication procedures for each seed stock used or novel genotype generated. Describe any experiments used to assess the effect of a mutation and, where applicable, how potential secondary effects (e.g. second site T-DNA insertions, mosiacism, off-target gene editing) were examined.*
