# Supplementary material for: Pathogenic variants reveal candidate genes for prostate cancer germline testing for men of African ancestry
Source: Nat Commun. 2025 Oct 2;16:8799. doi: 10.1038/s41467-025-63865-6 (PMC12491615; doi:10.1038/s41467-025-63865-6)
Supplement: Supplementary file 4 — Description of supplementary files [file 41467_2025_63865_MOESM4_ESM.pdf]

Description of Additional Supplementary Files

File name: Supplementary Data 1

Description: Genetic ancestral fractions for the 217 African ancestral prostate cancer (PCa) patients, defined for K = 3 and K = 4 population inference.

File name: Supplementary Data 2

Description: Single nucleotide variants (SNVs) identified as potentially pathogenic in 217 African ancestral prostate cancer patients.

File name: Supplementary Data 3

Description: Insertion/deletion (indel) variants identified as potentially pathogenic in 217 African ancestral prostate cancer patients.

File name: Supplementary Data 4

Description: Variants identified as potentially pathogenic among 959 PPCG European ancestral prostate cancer (PCa) patients.

Abbreviations: GT: germline Testing, AA: amino acid

File name: Supplementary Data 5

Description: Variants identified as potentially pathogenic among 3,209 European ancestral MGRB healthy individuals.

Abbreviations: GT: germline Testing, AA: amino acid

File name: Supplementary Data 6

Description: Potentially oncogenic variants in 217 African ancestral prostate cancer patients.

File name: Supplementary Data 7

Description: Rare Potentially Oncogenic Variants (POVs) identified in 217 African ancestral prostate cancer patients impacting 45 DNA Damage Repair (DDR) unknown candidate germline testing genes.

File name: Supplementary Data 8

Description: Population-specific low-frequency variants in 217 African ancestral prostate cancer patients.

File name: Supplementary Data 9

Description: List of the candidate variants and genes in 217 African ancestral prostate cancer patients.

File name: Supplementary Data 10

Description: Ranked list of genetic variants for germline testing panel in African prostate cancer patients.
